# Management of Forehead Dermatofibrosarcoma Protuberans With Wide Local Excision and Paramedian Forehead Flap Reconstruction: A Case Report

**DOI:** 10.7759/cureus.76322

**Published:** 2024-12-24

**Authors:** Ahmad Irshad, Muhammad Amar Akram, Sajid Ijaz, Abdur Rehman, Amna Khalid, Maryam Iftikhar

**Affiliations:** 1 General Surgery, Mayo Hospital, Lahore, PAK; 2 Plastic and Reconstructive Surgery, Mayo Hospital, Lahore, PAK; 3 Surgery, Mayo Hospital, Lahore, PAK; 4 Internal Medicine, Northampton General Hospital NHS Trust, Northampton, GBR; 5 Pathology, Margalla Institute of Health Sciences, Rawalpindi, PAK

**Keywords:** dermatofibrosarcoma protuberans, dfsp, forehead reconstruction, soft tissue sarcoma, wide local excision

## Abstract

Dermatofibrosarcoma protuberans (DFSP) is a rare, low to intermediate-grade soft tissue sarcoma that presents significant diagnostic and therapeutic challenges. We report the case of a 40-year-old male patient who presented with a slow-growing, asymptomatic lesion on his forehead that had developed over two years. Clinical examination revealed a firm, non-tender multinodular mass measuring 5 x 3 cm in the supraorbital region. The diagnosis was confirmed through histopathological examination and immunohistochemical staining, which demonstrated the characteristic storiform pattern and CD34 positivity. The patient underwent wide local excision with 3 cm margins, followed by reconstruction using a paramedian forehead flap. The procedure involved upper and lower eyelid release, lateral canthotomy, and canthoplasty to facilitate proper reconstruction. Postoperative recovery was satisfactory, though a subsequent procedure was required for further excision and secondary healing of a residual lesion. This case highlights the importance of early recognition, appropriate imaging, and the complex surgical approach often required for complete excision and reconstruction of DFSP in anatomically challenging areas. Long-term follow-up remains essential due to the high recurrence risk associated with this neoplasm.

## Introduction

Dermatofibrosarcoma protuberans (DFSP) is a rare, low to intermediate-grade soft tissue sarcoma of fibroblastic origin. Characterized by its slow growth and low metastatic potential, DFSP presents significant challenges in diagnosis and management due to its high rate of local recurrence [1]. This neoplasm typically affects young to middle-aged adults, with a peak incidence at 20-50 years of age [2]. Epidemiologically, DFSP has an estimated annual incidence of 0.8-4.5 cases per million in the United States, with a notably higher prevalence among African American individuals [3]. While predominantly occurring in adults, pediatric cases account for 6-20% of all DFSP diagnoses, including rare congenital presentations [4].

Clinically, DFSP most commonly manifests as an asymptomatic, indurated plaque that gradually develops into a multinodular mass. The tumor exhibits a predilection for the trunk, proximal extremities, and head and neck regions [5]. Its variable presentation, including potential atrophic variants, often leads to misdiagnosis or delayed diagnosis, underscoring the importance of clinical vigilance [6]. Histopathologically, DFSP is characterized by a storiform pattern of spindle cells infiltrating the dermis and subcutaneous tissues. The tumor displays a distinct immunohistochemical profile, typically positive for CD34 and negative for factor XIIIa, which aids in differentiating it from other fibrous and histiocytic tumors [7]. The identification of the characteristic t(17;22) translocation, resulting in *COL1A1-PDGFB* gene fusion, has not only enhanced diagnostic accuracy but also paved the way for targeted therapies [8].

Management of DFSP remains challenging, with wide local excision being the mainstay of treatment. However, high recurrence rates, even with adequate margins, have led to the exploration of alternative approaches such as Mohs micrographic surgery and molecular-targeted therapies [9]. This case report presents a patient diagnosed with DFSP, highlighting the clinical course, diagnostic challenges, and management strategies employed. By sharing our experience, we aim to contribute to the growing body of knowledge on DFSP and emphasize the importance of a multidisciplinary approach in managing this rare but potentially morbid neoplasm.

## Case presentation

A 40-year-old male patient presented with a slow-growing, asymptomatic lesion on his forehead. The patient had first noticed the lesion approximately two years prior to seeking medical attention. Initially, it appeared as a small, firm, raised area on the skin, which the patient had initially dismissed as inconsequential. Over time, the lesion gradually increased in size, prompting the patient to seek medical evaluation. He denied any recent weight loss, fever, night sweats, or chills. The patient's medical history was unremarkable, with no personal or family history of skin cancer or other malignancies.

**Figure 1:**
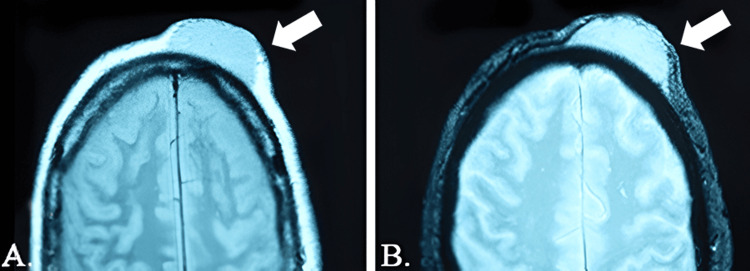
(A) Left side of forehead prominently occupied by DFSP, indicated by the arrow. (B) Nuclear magnetic resonance imaging of the solid mass showing no infiltration into the bone layer. DFSP: dermatofibrosarcoma protuberans

On physical examination, a firm, non-tender, multinodular mass measuring approximately 5 x 3 cm was observed on the forehead, specifically in the supraorbital region. The lesion was noted to be protuberant with a smooth surface. There was no evidence of ulceration, bleeding, or surrounding erythema. The overlying skin appeared slightly taut but intact. No palpable cervical or axillary lymph nodes were detected. Given the clinical presentation, a suspicion of DFSP was raised. A soft tissue ultrasound was performed, revealing a poorly defined, heterogeneous cutaneous tumor. Magnetic resonance imaging (MRI) demonstrated a heterogeneous lesion with peripheral enhancement, extending into the subcutaneous layer but without infiltration of the adjacent muscular or bony structures. The MRI was instrumental in preoperative planning by providing detailed visualization of the tumor's extent and margins. This imaging helped delineate the boundaries for wide local excision, ensuring adequate resection while preserving critical structures such as the orbital rim and associated musculature. Additionally, the absence of bony or muscular infiltration confirmed by MRI allowed the surgical team to plan a reconstruction approach without requiring additional procedures for structural repair. The lesion is illustrated in Figure 1.

**Figure 2:**
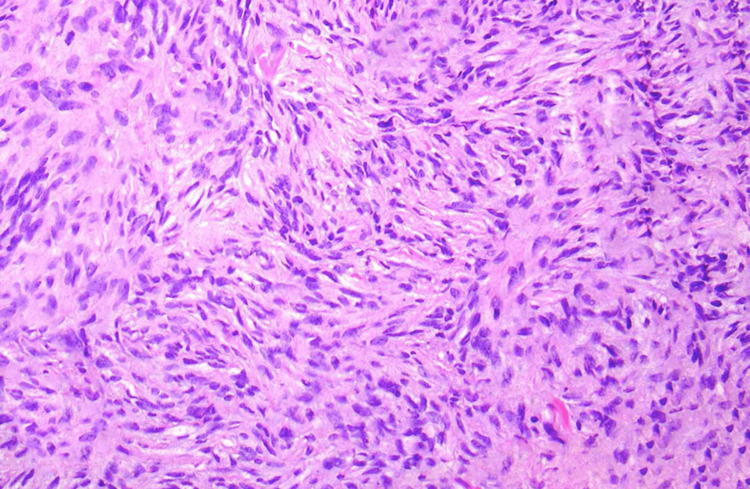
Histopathological examination of DFSP showing characteristic storiform pattern of spindle cells arranged in a cartwheel-like configuration. DFSP: dermatofibrosarcoma protuberans

A biopsy was performed, and histopathological examination revealed a cellular spindle cell neoplasm with vague cellular borders and relatively uniform elongated nuclei arranged in a storiform pattern. Immunohistochemical staining showed diffuse positivity for CD34 and vimentin while staining for smooth muscle actin, desmin, S100 protein, and keratins 8/18 was negative. These findings confirmed the diagnosis of DFSP (Figure 2).

**Figure 3:**
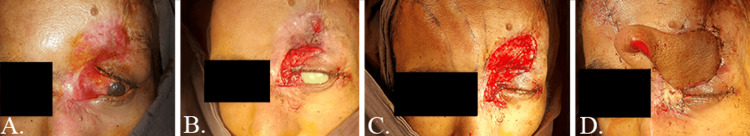
Sequential stages of surgical management. (A) Initial clinical presentation, (B) Post-initial excision, (C) Wide local excision defect, (D) Post-reconstruction with forehead flap.

Following the diagnosis, the patient was scheduled for wide local excision of the tumor with a 3 cm margin, followed by reconstruction using a paramedian forehead flap. The procedure was performed under general anesthesia and involved excision of the tumor, upper and lower eyelid release, and lateral canthotomy and canthoplasty to facilitate proper reconstruction (Figure 3).

Postoperatively, the patient recovered well and was closely monitored for flap viability and wound healing. Follow-up examinations showed good integration of the flap, though a subsequent procedure was required for further excision and secondary healing of a residual lesion in the left supraorbital region.

## Discussion

The case presented herein exemplifies the diagnostic and therapeutic challenges associated with DFSP, highlighting the importance of early recognition, appropriate imaging, histopathological confirmation, and the often complex surgical approach required for complete excision and reconstruction in anatomically challenging areas such as the forehead. The diagnosis often poses a significant challenge due to its indolent nature and variable clinical presentation. As observed in our case and corroborated by the literature, DFSP can manifest as a slow-growing, asymptomatic lesion that may be easily overlooked or misdiagnosed in its early stages [1]. The potential for atrophic variants, which can mimic benign conditions such as morphea, further complicates the diagnostic process [6].

Histopathological examination remains the gold standard for diagnosis, with the characteristic storiform pattern of spindle cells infiltrating the dermis and subcutaneous tissues being a hallmark feature [7]. The immunohistochemical profile, particularly CD34 positivity and factor XIIIa negativity, plays a crucial role in differentiating DFSP from other fibrous and histiocytic tumors [1]. In our case, these diagnostic criteria were instrumental in confirming the diagnosis of DFSP.

The management of DFSP has evolved significantly over the years, with WLE traditionally being the mainstay of treatment. However, the high recurrence rates associated with WLE, even with seemingly adequate margins, have led to the exploration of more precise surgical techniques [10]. MMS has emerged as a promising approach, offering improved local control while preserving healthy tissue. This technique is particularly valuable in anatomically sensitive areas where tissue conservation is crucial [11]. Our experience with MMS in this case aligns with recent literature suggesting superior outcomes in terms of local recurrence rates compared to traditional WLE [12].

The paramedian forehead flap was a pivotal component of the reconstructive strategy in this case, chosen for its robust blood supply and ability to provide excellent aesthetic and functional outcomes in the forehead region. The flap was designed based on the supratrochlear artery, which was carefully preserved during the elevation to ensure adequate perfusion. The pedicle was meticulously outlined to include a sufficient width and length of tissue for coverage of the defect while maintaining its vascular integrity. During the procedure, a lateral canthotomy and canthoplasty were performed to facilitate access and ensure optimal positioning of the reconstructed tissue. These techniques involved a precise incision at the lateral canthal tendon, followed by controlled soft tissue release to allow for greater mobility and alignment during reconstruction. Care was taken to avoid excessive tension on the flap, as well as to minimize trauma to the pedicle, to preserve its vascular supply. Additionally, intraoperative monitoring of flap viability, including visual inspection for adequate perfusion and color, was performed to ensure the success of the reconstruction. These technical considerations highlight the importance of a multidisciplinary approach and meticulous surgical planning in achieving satisfactory outcomes in cases requiring extensive excision and complex reconstruction [10-12].

Given the high recurrence risk associated with DFSP, a rigorous follow-up protocol is essential to monitor for local recurrence or new lesion development. For this patient, the follow-up plan included physical examinations every three months for the first two years, as recurrence rates are highest within this period. Dermatologic surveillance was emphasized, with a focus on inspecting the surgical site and surrounding tissue for any signs of recurrence. Annual imaging with MRI was planned to assess the deeper tissue layers and detect subclinical recurrences. In addition, the patient was advised to perform monthly self-examinations and report any new or suspicious changes at or near the surgical site. Unfortunately, the patient did not adhere to follow-up in the later stages of recovery, limiting our ability to provide long-term data on outcomes and recurrence in this case. This underscores the challenges in maintaining continuity of care and highlights the importance of robust patient education and support systems to ensure adherence to follow-up protocols [7].

In cases where complete surgical excision is challenging or in the presence of recurrent or metastatic disease, targeted therapy has shown promise. The identification of the characteristic t(17;22) translocation resulting in *COL1A1-PDGFB* gene fusion in most DFSP cases has paved the way for molecular-targeted therapies [8]. Imatinib mesylate, a tyrosine kinase inhibitor, has demonstrated efficacy in treating locally advanced or metastatic DFSP, offering a non-surgical option for select patients [9]. While not employed in our case, the availability of such targeted therapies represents a significant advancement in the management of complex DFSP cases.

While DFSP generally has a favorable prognosis with a low metastatic potential, the high risk of local recurrence necessitates long-term follow-up. Recurrence rates reported in the literature range from 0-60%, with most occurring within the first three years post treatment [13]. This wide variation underscores the importance of tailored follow-up protocols based on individual risk factors and treatment modalities employed. In our case, we have implemented a stringent follow-up regimen, emphasizing the importance of patient education regarding the need for regular check-ups and prompt evaluation of any new or changing lesions at or near the original tumor site.

As our understanding of DFSP biology continues to grow, there is a pressing need for multicenter studies to establish standardized treatment protocols and explore novel therapeutic approaches. The role of adjuvant radiotherapy in high-risk cases and the potential of immunotherapy in managing advanced DFSP are areas that warrant further investigation [14]. Additionally, the development of more sensitive and specific diagnostic tools, potentially incorporating molecular markers, could aid in earlier detection and more precise prognostication of DFSP. In cases where achieving clear surgical margins proves challenging, molecular-targeted therapies such as Imatinib, a tyrosine kinase inhibitor targeting the *COL1A1-PDGFB* fusion protein, may provide a valuable adjunct to surgery. This approach is particularly relevant in instances of residual disease, as in our case, where a second surgery was necessary. Incorporating such alternative strategies into treatment protocols could potentially reduce the need for repeated surgeries and improve overall patient outcomes.

## Conclusions

This case report highlights the complex nature of DFSP and the multidisciplinary approach required for its optimal management. The successful outcome in our patient underscores the importance of accurate diagnosis, appropriate surgical management, and diligent follow-up. As we continue to advance our understanding of DFSP biology and refine our treatment strategies, there is hope for improved outcomes in patients with this challenging tumor. Increased awareness among clinicians, coupled with advances in surgical techniques and targeted therapies, offers the potential for enhanced quality of life and reduced morbidity for individuals affected by DFSP.
